# Antimicrobial efficacy of *Lippia citriodora* natural extract against *Escherichia coli* and *Enterococcus faecalis* in “Piel de Sapo” melon juice

**DOI:** 10.1002/fsn3.1260

**Published:** 2019-11-10

**Authors:** Javier Rúa, Iván López‐Rodríguez, Javier Sanz, Pilar del Valle Fernández, Maria del Camino Garcia, Maria Rosario Garcia Armesto

**Affiliations:** ^1^ Department of Molecular Biology University of León León Spain; ^2^ Food Science and Food Technology Institute León Spain; ^3^ Department of Food Hygiene and Food Technology University of León León Spain; ^4^ Present address: mAbxience Parque Tecnológico de León León Spain

**Keywords:** Piel de sapo, antimicrobial activity, *Enterococcus faecalis*, *Escherichia coli*, *Lippia citriodora* extract, melon juice

## Abstract

**Background:**

The minimal inhibitory concentration (MIC) of an aqueous extract of *Lippia citriodora* with reported functional properties (PLX^®^) was determined on two strains of *Escherichia coli* (*E. coli*) belonging to serogroups commonly associated with foodborne illnesses (*E. coli* O157:H7 ATCC 700728 and *E. coli* O111 isolate 172) in vegetable products and two control strains for antimicrobial tests assays (*E. coli* ATCC 25922 and *Enterococcus*–*En. faecalis* ATCC 29212).

**Results:**

Mean MIC values at standard pH (7.4) in broth for the *E. coli* strains tested ranged from 4,444 µg/ml (35ºC) to 1,250 µg/ml (10ºC) and to 182 µg/ml (4ºC). At pH 5.5, conditions resembling those of melon juice, MIC was about 2 times higher at 35 and 10ºC compared with 4ºC. The MIC of *En. faecalis* was similar or slightly lower than those of *E. coli* at the conditions tested. In melon juice fortified with PLX^®^ (2,500 µg/ml, maximum sensorial acceptable limit), the three strains of *E. coli* maintained their viability although none showed growth potential after 4 days at 4ºC.

**Conclusions:**

PLX^®^ could be added to melon juice to control *E. coli* O157:H7 and *E. coli* O111 during refrigerated storage, reducing the risk of microbiological contamination in this food.

## Graphical abstract

In melon juice fortified with PLX® (2,500 µg/ml, maximum sensorial acceptable limit), the three strains of E. coli maintained their viability although none showed growth potential after 4 days at 4ºC. PLX® could be added to melon juice to control *E. coli* O157:H7 and *E. coli* O111 during refrigerated storage, reducing the risk of microbiological contamination in this food.

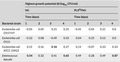

## 1 INTRODUCTION

Melon (*Cucumis melo* L.) is a widely cultivated crop consumed worldwide, with a global production in 2016 of about 31 million tons, most of which from China (ca 51%; FAO, 2018). Spain is included among the top ten melon‐producing countries in the world and is the first country exporting melons to the European Union (EU); FAO, 2018). The most widely consumed variety in the domestic market is “Piel de Sapo” (Escribano & Lázaro, 2009) included in *C. melo* L. var. *saccharinus* (Inodorus varietal group; Condés & Hoyos, 2008). This group of melons is characterized by its low‐calorie content, refreshing properties, and pleasant sweet taste, being consumed mainly as fresh fruit or a minimally processed product (e.g., fresh‐cut melon or fresh juice; Lim, 2012).

Fresh melon products (particularly from the Cantaloupe varietal group) emerged as a food safety concern in the United States in the 1990s (Castillo, Martínez‐Téllez, & Rodríguez‐García, 2014) because of their growing involvement in foodborne disease outbreaks (0.5 outbreaks per year during 1973–1991 to 1.3 during 1992–2011, considering only outbreaks linked to a single variety of melon; Walsh, Bennett, Mahovic, & Gould, 2014). Therefore, it could be expected that the increase in melon outbreaks could be even higher considering not only all notified outbreaks, but also including those caused by fruit salads in which various types of melon, and taking into account the limited shelf life of fresh melon products (Walsh et al., 2014).

Melons may, occasionally, become contaminated with pathogenic microorganisms (including verotoxin‐producing *Escherichia coli*‐VTEC) during the preharvest process, harvest, and postharvest treatments (EFSA (European Food Safety Authority), 2013; USDA‐FDA, 2018). These pathogens may, consequently, be transferred into the fresh products obtained from melon. Recently available data indicate that VTEC ranks second, after *Salmonella* spp., among the bacterial agents more frequently responsible for melon outbreaks in the United States during the period 1973–2011.

To date, as far as we know, there have been no VTEC outbreaks associated with low‐acid melon juice (pH > 4.6); however, other acidic fruit juices (pH ≤ 4.6), such as fresh apple juice/cider, have been linked to a number of serious incidents related to VTEC infection in the United States during the 1990s (mostly serotypes O157:H7 and O111) (see Salomão (2018)). Both serogroups are among the VTEC most frequently associated with severe illnesses in the United States and EU and are characterized by low infective dose (Croxen et al., 2013). As a result, fruit juices with pH > 3 have to be considered potential sources for pathogenic strains of *Enterobacteriaceae *(Reinders, Biesterveld, & Bijker, 2001). In order to control the presence of possible pathogenic microorganisms in fresh juices, refrigeration must be used as an additional barrier to the more or less acidic pH of nonpasteurized fruit juices (ICMSF, 2011). Furthermore, the application of naturally occurring antimicrobial compounds, such as herbal extracts, has recently been proposed to increase the safety of fresh juices (Shahbaz, Kim, Kim, & Park, 2018). These natural products could even improve shelf life and functional properties of the original juice. In this sense, we recently proposed the use of an aqueous extract of *Lippia citriodora* (PLX^®^) in melon juice (MJ) to improve its functional properties and antioxidant capacity (Rúa et al., 2018). For this, the aim of the presented research was to investigate the potential effect of PLX^®^ in inactivating *E. coli* and *En. faecalis* in broth under standard pH and temperature conditions, as well as those resembling refrigerated preservation of melon juices. Finally, we investigated the survival of these microorganisms in melon juice, fortified with PLX^®^ (PLX^®^FMJ) at 2,500 µg/ml, stored under ordinary market preservation conditions (4ºC for 4 days; Rúa et al., 2018).

## 2 MATERIALS AND METHODS

### 2.1 Bacterial strains and growth conditions

Two verotoxigenic *E. coli* strains (O157:H7 and O111) and two control strains for antimicrobial test assays (*E. coli* ATCC 25922 and *En. faecalis* ATCC 29212) were used in this study (Table 1). Stock cultures were maintained on Tryptic Soy Broth (TSB) with 50% (*v/v*) glycerol at −40ºC. Activation was made by transferring the cultures to TSB + 0.6% (*w/v*) yeast extract (TSBYE) (Oxoid Ltd.) and incubated according to instructions provided by Culture Collections and Klare et al. (2005) The purity of the strains recovered in TSBYE was checked by plating on TSA for *En. faecalis* (observing the specific morphology) and Tryptone Bile Agar with X‐glucuronide (TBX) for *E. coli* strains.

**Table 1:** Pathogenic and control strains used in this study, their sources, and other characteristics

Strain	Culture collection	Origin/isolation	Comments	Application	Growth conditions
*Escherichia coli* O157:H7	ATCC 700728	Undetermined	Nontoxigenic Absence of Shiga toxin genes confirmed by PCR	Quality control strain for BBL chromagar	37ºC; Nutrient Broth or Nutrient Agar
*Escherichia coli* O111, 172	Isolate	Raw ewes’ milk	Nonverotoxigenic Nonhemolytic Resistant to ampicillin, bacitracin, cloxacillin, penicillin G, sulfamethoxazole, and tylosin		
*Escherichia coli*	ATCC 25922, CECT 434	Clinical	Incorrectly cited in previous editions of this catalog as identical to NCTC 10418 Biotype 1	Food testing control culture, control strain; susceptibility testing; evaluation of Muller‐Hinton Agar	37ºC Trypticase Soy Agar Broth
*Enterococcus faecalis*	ATCC 29212	Urine	NS	Food testing control strain; susceptibility testing; evaluation of Mueller‐Hinton Agar	37ºC Trypticase Soy Agar Broth with defibrinated sheep blood

Abbreviations: ATCC, American Type Culture Collection; CECT, Colección Española de Cultivo Tipo; NS, Not specified.

### 2.2 Preparation of PLX^®^ stock solution

*Lippia citriodora* commercial extract (PLX^®^) (25% verbascoside) was from Monteloeder S.L. PLX^®^ stock solution (10 mg/ml) was prepared by dissolving the appropriate amount of PLX^®^ in ethanol at 40% (*v/v*). This solution was stored frozen (−40ºC) until use.

### 2.3 MIC and MBC assays

The minimum inhibitory concentration (MIC) of PLX^®^ against four strains was estimated using the antimicrobial microdilution assay described in ISO 10932:2010 ([ISO] International, 2010). Overnight cultures in TSBYE are diluted with sterile lab susceptibility test medium (LSM) (Klare et al., 2005) for *En. faecalis* and with sterile Mueller‐Hinton (pH 7.4) for *E. coli* and are plated in TSA to enumerate viable colonies and determine the appropriate dilution that corresponds to a concentration of 5.0 log_10_ CFU/ml (initial inoculum), according to ICMSF and International (1978).

Experiments were carried out in V‐shape‐bottomed 96‐well microplates incubated at 35 (24 hr), 10 (120 hr), and 4 º C (240 hr). The MIC value was considered to be the minimum concentration of antimicrobial compound that inhibits visible growth of the test strain (Barry, 1976). In another set of experiments, we used LSM broth or Mueller‐Hinton at pH value 5.5 by adjusting both broths with 1 mol/L HCl. Prior to the assay, the effect of the concentration of ethanol used was investigated, and we did not find any inhibitory effect of the growth of bacteria. Minimal bactericidal concentration (MBC) was determined by using aliquots from wells corresponding to the MIC values and from those with higher concentration and observing the lacking of growth on TSA. MBC was defined as ≥ 99.9% (3 log_10_) decrease in viable cells (Barry, 1976). At least two independent tests were performed in duplicate with each strain.

### 2.4 Survival and growth of *E. coli* and *En. faecalis* in “Piel de Sapo” melon juice at 4 º C

Melon juice was prepared as we have previously reported (Rúa et al., 2018). Freshly nonsterilized extracted MJ (25 ml) and PLX^®^FMJ at a final concentration of PLX^®^ of 2,500 µg/ml were inoculated separately with 10^3^ CFU/ml for *E. coli* and 10^5^ CFU/ml for *En. faecalis*. Survival and growth of the strains were assessed at 0, 1, 2, 3, and 4 days at 4ºC. At each sampling time, aliquots of 1 ml were taken, diluted in peptone water (0.1% *w/v*), and plated on TSA + 0.6% yeast extract (YE) (*w/v*) (TSAYE) (for *En. faecalis*) and on Tryptone Bile Agar with X‐glucuronide, Biokar (TBX agar), for *E. coli* for enumeration. As the TSBYE medium for *En. faecalis* is not selective, the counts were corrected for the possible presence of this bacterium in MJ, using a control of juice without inoculation and taking into account the morphology of this bacterium. The plates were incubated for 24 hr at 35ºC, and the results were presented as log_10_ CFU per milliliter of juice. The experiments were done in two batches of melon juice, each consisting of two conditions (MJ and PLX^®^FMJ). In each condition included within the batch, samples were taken in duplicate each day to evaluate microbial growth. For each strain and for each day of storage, the difference between the log_10_ CFU/ml at the evaluation day and the log_10_ CFU/ml at the beginning of the experiment (t = 0) was calculated for the two lots. Growth potential (δ) was defined as the highest value obtained between two lots. The results were interpreted considering that a value δ > 0.5 log_10_ indicates that the melon juice is able to support the growth of the bacteria tested (Beaufort, Cornu, Bergis, Lardeux, & Lombard, 2014).

### 2.5 Statistical analysis

All data analyses were performed using the SPSS 24.0 package (SPSS software available at the University of León). One‐way ANOVA with Tukey's multiple comparison test was used for the analysis of parametric data.

## 3 RESULTS AND DISCUSSION

### 3.1 Antimicrobial activity of PLX^®^ against *E. coli* and *En. faecalis* in broth under different temperature and pH conditions

The antibacterial activity (MICs and MBCs) of PLX^®^ for the four strains studied (Table 1) was evaluated in broth media at three temperatures and two pH values. The selected temperatures were as follows: 35ºC (optimum), 10ºC (abuse), and 4ºC (refrigeration), and the pH values were as follows: 7.4 for *E. coli* and 6.7 for *En. faecalis* (standard broth) and 5.5 (pH resembling that of the melon juice). In each one of the tested conditions of pH and temperature, the homogeneity of the MIC values obtained for the tested strains was especially noteworthy, mainly for the three strains of *E. coli*, as statistically (*p* < .05) they are included in one group (Table 2). On the other hand, we detected differences in the inhibition of the four bacteria, obtaining the highest MIC values (Table 2) at 35°C/pH 5.5 for all strains (between 7,500 and 10,000 µg/ml) and the lowest (≤78.12 µg/ml) at 4ºC/pH 5.5. At the abuse temperature (10ºC), for all strains we obtained MICs closer to those of 35ºC for the two pH values.

**Table 2:** Antibacterial activity (MIC) of PLX^®^ (µg/ml) against the strains used in this study under the conditions indicated^a^

Bacteria tested	Minimum inhibitory concentration (MIC) (µg/ml)					
	35ºC/std pH	35ºC/pH 5.5	10ºC/std pH	10ºC/pH 5.5	4ºC/std pH	4ºC/pH 5.5
*Escherichia coli* O157:H7	4,375.00 ± 1,157.28^a^	10,000 ± 0.00^a^	1,250 ± 0	2,500.00 ± 0.00^a^	195.31 ± 78.12^a^	≤78.12^b^
*Escherichia coli* O111	4,375.00 ± 1,157.28^a^	10,000 ± 0.00^a^	1,250 ± 0	2,500.00 ± 0.00^a^	195.31 ± 78.12^a^	≤78.12
*Escherichia coli* ATCC 25922	4,583.33 ± 1,212.68^a^	10,000 ± 0.00^a^	1,250 ± 0	3,750.00 ± 1,433.77^a^	156.25 ± 0.00^a^	≤78.12
*Enterococcus faecalis*	3,352.27 ± 1953.18^a^	7,500.00 ± 2,672.61^b^	1,250 ± 0	2,500.00 ± 0.00^a^	312.50 ± 0.00^b^	≤78.12

a Data are expressed as means ± *SD*. In each column, different letters mean significant differences (*p* < .05). 24 hr of culture at 35ºC, 120 hr of culture at 10ºC, and 240 hr of culture at 4ºC.

b It is the lowest concentration used in the assay. std pH, standard pH (7.4 for *Escherichia coli* or 6.7 for *Enterococcus faecalis*).

It is remarkable that PLX^®^ MICs were lower at 4ºC than at the other two temperatures for all strains tested, regardless of the assay culture broth pH (standard or 5.5). Particularly, at pH 5.5, MIC was about half that of MIC at standard pH for all *E. coli* strains but about a quarter the MIC at standard pH for *En. faecalis* (Table 2). This indicates that the four bacteria tested are less tolerant to the more acidic pH at lower temperature (4°C), similar to what we observed in a previous study (Rúa et al., 2018) with two probiotic‐type lactic acid bacteria (PT‐LAB) (*Lactobacillus delbrueckii* subsp. *bulgaricus* and *Streptococcus salivarius* subsp. *thermophilus*), whose PLX^®^ MIC values were also ≤ 78.12 μg/ml. It has been described that the refrigeration temperature has a specific influence on the MICs of NaCl and sodium lactate for different spoilage organisms and pathogens, including *En. faecalis* but not *E. coli. *(Houtsma, Kant‐Muermans, Rombouts, & Zwietering, 1996) Belda‐Galbis, Leufvén, Martínez, and Rodrigo (2013) reported that a decrease in temperature (from 35 to 15 or 8ºC) produces a delay and lower growth in *E. coli* K12, irrespective of the antimicrobial used.

The mean MBC values for each temperature and pH values were higher than the mean MIC values corresponding to the same conditions, except that the MBC for PLX^®^ was equal to its MIC at 35ºC/ pH 5.5 for three strains of *E. coli* (Table 3). In addition, MBCs were slightly higher at pH 5.5 than at standard pH for *E. coli* ATCC 25922 and *En. faecalis*, while for *E. coli* O157:H7 and *E. coli* O111 MBCs were equal at 35ºC or slightly lower at 10 and 4ºC. At pH 5.5 and the three temperatures, there were no significant differences among the four strains tested, which seems to indicate a more homogeneous antimicrobial effect of PLX^®^ at pH resembling of melon juice. Taking into account the general consideration (differences in MIC and MBC values not more than twofold (Moody & Knapp, 2007), the effect for PLX^®^ is mostly bacteriostatic for all strains under the conditions studied, with the exception of 35ºC/pH 5.5 for all strains and 35ºC/standard pH for *E. coli* O157:H7 and *E. coli* O111 for which the effect is bactericidal. So, it seems that the bactericidal action of PLX^®^ against the studied strains was not dependent on the type of microorganism, but on storage temperature.

**Table 3:** Bactericidal activity (MBC) of PLX^®^ (µg/ml) against the strains used in this study, under the conditions indicated^a^

Bacteria tested	Minimum bactericidal concentration (MBC) (µg/ml)					
	35ºC/std pH	35ºC/pH 5.5	10ºC/std pH	10ºC/pH 5.5	4ºC/std pH	4ºC/pH 5.5
*Escherichia coli* O157:H7	10,000 ± 0.00^a^	10,000 ± 0.00	10,000 ± 0.00^a^	8,333.33 ± 2,581.98^a^	10,000 ± 0.00^a^	8,333.33 ± 2,581.98^a^
*Escherichia coli* O111	10,000 ± 0.00^a^	10,000 ± 0.00	10,000 ± 0.00^a^	8,333.33 ± 2,581.98^a^	10,000 ± 0.00^a^	8,333.33 ± 2,581.98^a^
*Escherichia coli* ATCC 25922	7,500 ± 2,594.37^a,b^	10,000 ± 0.00	7,272.72 ± 2,783.93ª^,b^	8,333.33 ± 2,581.98^a^	7,272.72 ± 2,783.93ª^,b^	8,333.33 ± 2,581.98^a^
*Enterococcus faecalis*	5,625 ± 2,677.07^b^	10,000 ± 0.00	5,625 ± 2,677.07^b^	8,333.33 ± 2,581.98^a^	5,625 ± 2,677.07^b^	8,333.33 ± 2,581.98^a^

a Data are expressed as means ± *SD*. In each column, different letters mean significant differences (*p* < .05). 24h of culture at 35ºC, 120 hr of culture at 10ºC, and 240 hr of culture at 4ºC. std pH, standard pH (7.4 for *Escherichia coli* or 6.7 for *Enterococcus faecalis*).

The effect of antimicrobials on a microorganism depends on several factors, such as pH and temperature. The pH influences in the interaction of the phenolic compound with the membrane of the microorganism or in the dissociation of the molecule to be more effective; also, in general, microorganisms are more resistant to all kind of treatment at their optimum pH, but at lower or higher pH the sensitivity is increased (Adams, 2014; Jay, 1992; Mackey, Forestière, & Isaacs, 1995). The temperature of the medium in which the microorganism is suspended determines the fluidity of the membrane, for example, at low temperatures phospholipids in bacterial cell membranes are closely packed while at high temperatures they are more disorderly arranged. As explained by Aronsso and Rönner (2001), this could explain why the bactericidal action of an antimicrobial is better at high temperatures.

Verbascoside, the main component of PLX^®^, can establish interactions with phospholipids in model membranes at pH 7.4, but not at a low pH (*i.e.,* at pH 3.0) (Funes, Laporta, Cerdán‐Calero, & Micol, 2010); therefore, the interaction of the phenolic compound with the membranes could contribute to the antimicrobial mode of action of verbascoside. In our study, standard pH (6.7 or 7.4) is close to the effective pH of the verbascoside–phospholipid interaction, which could partly explain the greater antimicrobial effect of PLX^®^ (MICs) at this pH (at 35 and 10ºC for all strains already tested).

On the other hand, PLX^®^ needs time to enter into bacterial cells through cell membranes and damage them. This is the reason for similar antibacterial action (MBCs) of PLX^®^ at the three temperatures, with different times (1 day at 35ºC, 5 days at 10ºC, and 10 days at 4ºC). Higher MIC and MBC values have been reported for PLX^®^ against *E. coli* CECT 515 (12,800 µg/ml and 51,200 µg/ml, respectively, in TSB at 37ºC after 24h, using a microdilution technique) (Giner et al., 2012). However, Kumar, Kumar, Raman, and Reddy (2008) estimated lower MIC values (10–100 µg/ml) for acetone extract of *Lippia citriodora* against *E. coli* ATCC 1175, using an agar well diffusion method, and Bazzaz, Klameneh, Ostad, and Hosseinzadeh (2018) reported a MIC value > 200 µg/ml for lemon verbena extract against two isolated strains of *E. coli*. With regard to *En. faecalis*, the MIC and MBC values obtained in this study are very similar to those previously reported for other PT‐LAB (Rúa et al., 2018) and *Bacillus cereus*: 3,200 µg/ml (MIC) and 6,400 µg/ml (MBC) (Giner et al., 2012), but ten times higher than those reported for *Staphylococcus aureus* (MIC and MBC of 400 µg/ml and 800 µg/ml, respectively; Giner et al., 2012).

The lower MIC and MBC values of *En. faecalis* in comparison with *E. coli* strains in some growth conditions could be due to the differences in cell wall composition of Gram‐negative and Gram‐positive bacteria (Nikaido & Neidhardt, 1996; Nikaido, 2003). In the same way, *E. coli* was more resistant to malic acid (MBCs three times higher) than *Listeria monocytogenes* at 5ºC than at 20 or 35ºC for 24 hr in mango, pineapple, and papaya juices (Rathnayaka, 2013).

### 3.2 Viability of *E. coli* and *En. faecalis* in refrigerated “Piel de Sapo” melon juice

When the three strains of *E. coli* and the strain of *En. faecalis* were grown in both MJ and PLX^®^FMJ (Rúa et al., 2018) (Table 4), we only detected growth potential (‐δ –>0.5) in *En. faecalis* strain after 4 days of refrigerated storage at 4 º C. However, we neither detected growth potential of the three *E. coli* strains in PLX^®^FMJ nor MJ in these conditions. These data indicate that only the *En. faecalis* strain has a great capacity to resist adverse or stress factors, such as phenolic compounds present in PLX^®^ combined with low storage temperature (4ºC). In a previous study, we reported that MJ was a suitable medium of growth for four of the six tested PT‐LAB and that PLX^®^FMJ allows the growth of *Lb. rhamnosus* GG (at 4ºC for 4 days; Rúa et al., 2018).

**Table 4:** Highest growth potential (δ) (log_10_ CFU/ml) among the batches for *Escherichia coli* and *Enterococcus. faecalis* strains used in this study, in “Piel de Sapo” plain melon juice and fortified with PLX^®^ (2,500 µg/ml) stored for 4 days at 4ºC

Bacterial strain	Highest growth potential (δ) (log_10_ CFU/ml)							
	MJ				PLX^®^FMJ			
	Time (days)				Time (days)			
	1	2	3	4	1	2	3	4
*Escherichia coli* O157:H7	−0.05	0.05	0.21	0.25	−0.45	0.05	0.21	0.0
*Escherichia coli* O111	−0.22	0.08	−0.49	0.33	0.04	0.25	0.13	0.0
*Escherichia coli* ATCC 25922	0.13	0.16	**0.56**	0.27	0.13	−0.07	0.23	0.33
*Enterococcus faecalis*	**0.54**	0.32	0.41	**0.65**	0.49	0.28	0.49	**0.87**

Note

δ ≤ 0.5 log_10_, the melon juice is not able to support the growth of the bacteria. δ > 0.5log_10_, the melon juice is able to support the growth of the bacteria (in bold). Growth potential (log_10_ CFU/ml) is estimated as the difference in the media of results at one day of storage at 4ºC and the media of the results at the onset of the storage (0 days).

Abbreviation: MJ, plain melon juice; PLX^®^FMJ, plain melon juice fortified with PLX^®^.

Melons of the Inodorus varietal group, which the variety “Piel de Sapo” belongs to, contain a wide range of sugars including sucrose, glucose, and fructose (Amaro, Oliveira, & Almeida, 2015). However, culture media contain no sugars (Mueller‐Hinton) or the content is lower (LSM) which can justify the growth of *En. faecalis* in melon juice at a concentration of PLX^®^ above the MIC obtained in the LSM broth at refrigeration temperature. It would be interesting to check the growth of *En. faecalis* and *E. coli* in juices obtained from other melon types with different metabolizable sugar composition ( Amaro et al., 2015).

The effectiveness of natural antimicrobials, such as plant‐derived compounds, has been demonstrated against foodborne pathogens (Holley & Patel, 2005; Tajkarimi, Ibrahim, & Cliver, 2010). Therefore, the increased occurrence of these microorganisms could serve as motivation to find effective natural antimicrobials to use as food preservatives. The results of the present study indicate that *Lippia citriodora* extract (PLX^®^) might be used as an alternative hurdle to reducing the risk associated with foodborne pathogen infection in fruit juice.

## 4 CONCLUDING REMARKS

The antimicrobial effect of PLX^®^ in broth is most effective at low temperatures (10 and 4°C) at both pH values (standard and resembling those of melon juice) for all strains tested, including the two strains of *E. coli* belonging to serogroups commonly associated with foodborne illnesses. In addition, for these two pathogen strains, a bactericidal effect of PLX^®^ was observed at 35°C at both pH values. On the other hand, *En. faecalis*, but not the three strains of *E. coli*, in PLX^®^FMJ, was able to survive/grow at concentrations of PLX^®^ higher than the MIC in broth under the same pH (5.5) and temperature (4ºC) conditions. These results emphasize the need to check the effect of antimicrobial activity of plant extracts, such as PLX^®^, on the food itself to assess possible interaction with other intrinsic factors of the food (*e.g.,* composition in sugars and vitamins). This work also underlines the importance of performing an individualized study of the spoilage and pathogen strains to determine their growth variability in food.

## CONFLICT OF INTEREST

The authors declare no conflict of interest.

## ETHICAL APPROVAL

This study does not involve any human or animal testing, and written informed consent was obtained for all study participants.
